# *Staphylococcus aureus *and Chronic Airway Disease

**DOI:** 10.1097/WOX.0b013e3181ecd8ae

**Published:** 2010-08-15

**Authors:** Lara Derycke, Claudina Pérez-Novo, Koen Van Crombruggen, Marie-Noëlle Corriveau, Claus Bachert

**Affiliations:** 1 Upper Airway Research Laboratory (URL), Department of Oto-Rhino-Laryngology, Ghent University Hospital, Ghent University, Belgium; 2 Centre Hospitalier Universitaire de Québec, Pavillon Saint-François d'Assise, Québec, Canada

**Keywords:** chronic rhinosinusitis, nasal polyps, *Staphylococcus aureus*, innate and adaptive immune system, superantigens

## Abstract

*Staphylococcus aureus *(*S. aureus*) is correlated with the development of persistent severe inflammatory disease of the upper airway including chronic rhinosinusitis with nasal polyps. This inflammation of the upper airways is characterized by a T-helper 2-driven disease: interleukin-5 is significantly increased and local production of immunoglobulin E is observed. *S. aureus *and its enterotoxins are deregulating the tissue inflammation at different levels: structural cells and the innate and adaptive immune system. Knowing the triggers of the pathomechanisms involved will greatly help us to find new therapeutic approaches to resolve this chronic inflammatory process.

## Introduction

Chronic rhinosinusitis (CRS) is an inflammatory process of the nose and sinuses that is characterized by nasal blockage/obstruction/congestion, nasal discharge, facial pain, and/or reduction of smell [1]. The diagnosis is based on symptoms and duration of the disease, nasal endoscopy, and CT scan. Rhinosinusitis lasting less than 12 weeks is in most cases of viral origin; however, it is currently unknown whether recurrences of this process may lead to chronic rhinosinusitis. CRS may be differentiated in CRS without nasal polyps and with nasal polyps (CRSwNP), the latter being characterized by a T_H_2-driven inflammatory process with increased levels of interleukin (IL)-5, infiltration of eosinophils, eosinophil cationic protein (ECP), and local immunoglobulin (Ig)E production. In severe cases asthma comorbidity and the presence of IgE against *Staphylococcus aureus *(*S. aureus*) enterotoxins (SAE-IgE) is frequently found [2]. Colonization rates of *S. aureus *in healthy subjects and patients with CRS have been found to be around 33.3 and 27.3%, respectively, whereas 63.6% of all subjects with CRSwNP were colonized with *S. aureus *[3]. By analysis of biopsies of sinus mucosa from CRS patients, it was also found that the presence of bacterial biofilm was increased compared with control patients [4]. Biofilms are formed when bacteria adhere to surfaces in an aqueous environment and begin to excrete a slimy, gluelike substance that can anchor them to tissue. A biofilm can be formed by a single bacterial species, but more often biofilms consist of multiple bacterial strains. *S. aureus *is one of the bacteria frequently present in biofilms within the nose, but it remains to be confirmed whether biofilms via *S. aureus *carriage contribute to the immune changes typical for nasal polyp disease.

## *S. Aureus *in the Nose

The Gram-positive bacterium *S. aureus *is a major pathogen in both community-acquired and nosocomial infections. *S. aureus *often colonizes the host asymptomatically and lives as a commensal in the human nose. The anterior nares are the major reservoir of *S. aureus*: 20% of the peoples are persistently colonized and 60% are intermittent carriers, whereas 20% never carry *S. aureus *[5,6]. The anterior nares are lined by a fully keratinized epidermis with hairs, sebaceous glands, and sweat glands. The vestibule is limited above and behind by a ridge, the limen nasi, over which the skin becomes continuous with the nasal mucous membrane. Apparently, the staphylococcal cells flourish here in the relative absence of human defenses and/or are capable of withstanding the local antibacterial defenses. To adhere, bacterial cells need to establish firm interactions with human cell surfaces to prevent their rapid elimination by physicochemical mechanisms. To establish successful colonization, it is thought that surface components of the staphylococcal cell interact with complementary components on the eukaryotic host cell membranes. Eukaryotic surface glycoproteins and proteoglycans that are present on the mucous membranes contribute to the adhesion of bacteria. *S. aureus *seem to adhere to mucincoated cells much better than to cells without such carbohydrate coating. Other substances like secretory immunoglobulin A, glycolipids, and surfactant protein A may also constitute receptor sites for *S. aureus *[7]. *S. aureus *strains can be divided into 4 major groups based on the *accessory gene regulator *(*agr*) locus. This locus controls the expression of most virulence factors, staphylococcal toxic shock syndrome toxin-1 producing isolates belong to agr specificity group III, and agr groups I and II are associated with enterotoxin-mediated diseases. *S. aureus *enterotoxins (SAE) are potent molecules called superantigens[8] that have the ability to simultaneously bind the invariant region of the MHC-II (major histocompatibility complex, class II) molecules on antigen-presenting cells and the T-cell receptor variable region *β *(TCR-V*β*) [9]. This leads to a potent activation and proliferation of T cells, which induces the synthesis of IgE by B cells and have direct effects on pro-inflammatory cells. In a study published by Van Zele et al,[10] it was found that 75% of the strains present in human nasal mucosa produced at least one enterotoxin. The enterotoxin gene cluster was observed in 67.5% of the strains, whereas the classic enterotoxins appeared only in 42.5% of the strains. With use of the PNA FISH method for the detection of intramucosal *S. aureus *in healthy subjects and in patients with CRS, significantly more *S. aureus *were observed in CRSwNP patients with aspirin-exacerbated respiratory disease (AERD) versus controls and CRS without nasal polyps [11].

## Effect of *S. Aureus *or SAE on the Innate Immune Response

Staphylococcal superantigens induce migration and maturation of dendritic cell (DC) populations in vivo. However, in contrast to lipopolysaccharides, superantigens failed to induce DC maturation in Recombination Activation Gene (RAG) or MHC class II-deficient mice, suggesting that T-cell activation was a prerequisite for DC maturation. The DC-activating properties of T cells were confirmed by the analysis of in vivo responses to nonmicrobial T-cell mitogens, suggesting that polyclonal activation of T cells in vivo may lead to activation of the innate immune response [12]. In an in vitro model where human monocyte-derived DCs were stimulated with *S. aureus *enterotoxin B (SEB), the authors demonstrated that SEB induces the maturation of DCs and initiates the secretion of high levels of IL-2 but not of IL12p70. The SEB-induced maturation could be blocked using anti-toll-like receptor 2 (TLR2) antibodies and anti-nucleotide oligomerisation binding domain 1 (NOD1) antibody or RNA interference for these receptors [13,14]. Furthermore, SEB-activated DCs were able to drive polarization of naive T cells into the T_H_2 subsets by upregulation of T-cell immunoglobulin mucin domain 4 (TIM4), as SEB-induced DC could interact with TIM1 receptor on T_H_2 cells. Thus, SEBs clearly shift DC to maturate and stimulate T_H_2 cells in a TLR2-dependent manner. FACS analysis of the different human DC subsets in CRSwNP tissue showed that the ratio of mDC to pDC was significantly decreased[15] compared with control tissue and furthermore DCs were more mature in CRSwNP (unpublished data). These findings suggest that DCs are implicated in the development of CRSwNP but further research is needed to clarify the direct impact of SAE on DC.

Peptidoglycan (PGN), a major component of Gram-positive bacteria cell walls and a potent activator of the mammalian immune system, activates cells through NOD2, PGN recognition proteins, and potentially TLR2/TLR6 heterodimers [16]. PGN can also stimulate cells indirectly by activating complement products like C3a and C5a. Indeed, these complement products are increased in nasal secretion of people suffering from CRSwNP [17]. C3a and C5a may induce mast cell degranulation leading to the release of histamine, and the activation of mast cells seems to be important for the accumulation of DC from the lymph nodes [18].

Not only DCs but also other innate immune cells such as mast cells or macrophages in the upper airways are activated by *S. aureus *or SEB. Mast cells are well-established effectors in allergic airway inflammation. Cross-linking of Fc*ε*RI by IgE-bound inhaled antigen can trigger degranulation defined by the release of the preformed secretory granule complex and subsequent extracellular dissociation of preformed mediators (eg, histamine and certain proteases). Activation is accompanied by the rapid synthesis of lipid mediators (eg, cys-LTs, dihydroxy leukotrienes, and prostaglandin D2), and the induction of cytokines and chemokines (eg, IL-4, IL-13, IL-12, IL-1, IL-18, and tumor necrosis factor-*α *[TNF-*α*]). Mast cell mediator release can also be triggered by innate signals, and innate mast cell activation can be part of a protective immunity to pathogens [19]. Mast cells may play an important role in CRSwNP, as abundant degranulated mast cells were observed in tissue sections. After *S. aureus *infection, mast cells increase the amount of TLR2 receptors on their surface and consequently release of TNF-*α *and IL-8 [20]. PGN, lipoproteins, and lipoteichoic acid from *S. aureus *are able to stimulate mast cells directly via the binding of TLR2 on these cells. Short stimulation of nasal tissue explants with Lipoteichoic Acid (LTA) or Surface Protein A (SPA) induced a significant increase in mast cell mediators, including histamine Leukotriene C_4_/D_4_/E_4 _(LTC_4_/D_4_/E_4_), and prostaglandin D_2 _(PGD_2_) [21]. Macrophage mannose receptor, an innate pattern recognizing receptor expressed by macrophages, is capable of phagocyte invaders (pathogens). The expression of macrophage mannose receptor is significantly upregulated in CRSwNP compared with turbinate tissue of controls [22]. Recent research by our group observed via immunohistochemistry and FACS analysis a significant upregulation of M2 macrophages in CRSwNP (unpublished data)*. S. aureus *inhibits the production of superoxide in macrophages to evade killing after phagocytosis; this mechanism is TLR2 mediated [23]. Cumulatively, these findings suggest that *S. aureus *is triggering a wide range of innate immune cells.

## Effect of *S. Aureus *or SAE on the Adaptive Immune Response

Nasal polyps (CRSwNP) showed increased numbers and activation of T cells, and an increase in plasma cells. Additionally, significantly higher levels of eosinophilic markers (eosinophils, eotaxin, and ECP) together with a T_H_2 polarization with high IL-5 and IgE concentrations have been found when compared with control nasal mucosa [24]. Furthermore, CRSwNP is characterized by a low concentration of transforming growth factor-*β*1 (TGF-*β*1), a downregulation of FOXP3 (transcription factor for T regulatory cells) and on transcriptional level with an upregulation of GATA-3 and T-bet compared with control nasal mucosa [25]. These results suggest that T_H_2 cells are dominant and block the development of T regulatory cells, or that there is a deficiency in T regulatory cells which allows the T_H_2 cells to dominate as a consequence of the lack of counter regulation. We need to be careful in generalizing this observation because nasal polyps from Asian patients are also characterized by T-cell activation and impaired regulatory T-cell function. However, in contrast to those from white patients, samples from Asian counterparts demonstrated a T_H_1/Th17 polarization [26]. So, what is the difference between these patients: trigger? innate response? Superantigens are shown to activate T cells and to cross-link MHC-II on antigen-presenting cells and the T-cell receptor variable region *β *chain on T cells. The observation of IgE antibodies to SAE in nasal polyp tissue homogenates demonstrated for the first time that these enterotoxins are involved in the pathogenesis of nasal polyp disease [27,28]. However, the prevalence of SAE-IgE formation differs between white (European) and Asian patients (unpublished data). Furthermore, IgA and IgG concentrations were significantly higher in tissue homogenates, but not in serum, of CRSwNP compared with CRS without nasal polyps and control subjects. CRSwNP with specific IgE to SAEs showed significantly higher concentrations of IgG and IgE antibodies, and this goes together with an increase in plasma cells (CD138^+^) found in CRSwNP tissue. This suggests a local production of these immunoglobulins in CRSwNP, likely in response to a chronic microbial trigger [29]. Furthermore, immunohistochemical staining for IgE, CD3, CD20, and CD138 demonstrated the presence of lymphoid accumulations and follicle-like structures in nasal polyp tissue, which suggests local IgE production [30]. There is even evidence that components (protein A) of the bacteria cell wall of *S. aureus *could trigger B-cell activation directly [31,32]. Stimulation of nasal tissue explants originating from inferior turbinate or nasal polyp tissue with the superantigen SEB for 24 hours stimulated the release of T_H_1 and Th 2 cytokines (IFN-*γ*, IL-2, IL-4, IL-5, IL-10, and IL-13). Furthermore, nasal polyp tissue released more cytokines after stimulation compared with control tissue [21].

In a mouse model, our group recently observed that SEB could facilitate the sensitization of CD4 cells to nasally applied allergen, resulting in the development of experimental asthma. Treatment with anti-CD4 antibodies could abrogate the development of allergy. To evaluate the contribution of DCs, nasal application of SEB was combined with FITC-labeled ovalbumin (OVA) that was administered intratracheally in the mice; DC migration toward the draining lymph nodes was observed after 24 hours and the maturation marker, CD86, was expressed at a higher level in OVA/SEB-treated mice compared with OVA/saline-treated mice [33]. Collectively, these data suggest that SAEs have strong effects on the proliferation of T cells.

## Effect of *S. Aureus *or SAE on Nonimmune Cells

Histomorphologic analysis of nasal polyps showed the presence of eosinophils, and the formation of a subepithelial cap over a pseudocyst area that was filled with albumin. TGF-*β *plays a crucial role in airway inflammation and remodeling. Furthermore, TGF-*β *is considered a master switch in the induction of the profibrotic program, and acts as chemoattractant and proliferation factor for fibroblasts. In CRSwNP the TGF-*β*1 signaling pathway is strongly downregulated and this coincides with lower collagen content in the polyp compared with control nasal mucosa [34]. Does *S. aureus *or SAE provoke changes in airway epithelial cells or fibroblasts? The airway epithelium not only is a physiological barrier but also actively is involved in the immune response and a major source of inflammatory cytokines and mediators. Nasal epithelial cells detect *S. aureus *through TLR2. The nasal mucosa can also respond directly to bacterial challenge through the elaboration of cationic polypeptides. Defensins are such cationic antibacterial peptides and secreted by epithelial cells. In humans 3 *β *defensins (HBD1, HBD2, and HBD3) are expressed; HBD2 has been shown to be upregulated upon infection and can also be induced upon TLR2 activation and chemotactically attract neutrophil to the site of infection. *S. aureus *and also other bacteria are capable of upregulating TLR2 expression on epithelial cells; however, when epithelial cells were exposed to *S. aureus*, TLR2 upregulation was delayed by 4 hours, and because of this delay, the expression of HBD2 was prevented [35]. Collectively, these data suggest that *S. aureus*/SAE probably suppresses the innate epithelial host response long enough to enable colonization of the nasal mucosa. Not only the epithelial cells but also the involvement of the fibroblasts in the pathogenesis of airway disease is increasingly acknowledged. Indeed, SEB challenge induced migration and blocked proliferation of pretreated IFN-*γ *fibroblasts isolated from inferior turbinate. IFN-*γ *was necessary to induce the expression of MHC-II molecules [36]. SEB treatment of fibroblasts decreased the expression of cyclooxygenase-2 (COX-2) and prostaglandin E_2 _(PGE_2_). This finding supports the idea that the presence of SEB may induce airway inflammation by downregulating the production of anti-inflammatory mediators.

## Role of *S. Aureus *or SAE in the Development of Asthma and AERD

The coexistence of asthma and CRS has been noted for a long time; however, the debate remains as to whether CRS is a risk factor for asthma. It seems that CRS and asthma are linked by a common inflammatory pathway among which eosinophils and airway epithelium play an important role. In many patients with severe asthma, CRSwNP is common and the presence of SAE has been recently identified as a possible link between these 2 diseases, resulting in severe disease manifestations in both upper and lower airways (unpublished data). Surgical treatment of CRS or nasal polyposis might improve asthma symptoms [37-39]. Furthermore, a recent study in teenagers proved the presence of IgE against SAE in atopic subjects, which was associated with asthma risk [40].

AERD is a clinical syndrome associated with chronic severe inflammation in the upper and lower airways, resulting in chronic rhinitis, sinusitis, recurrent polyposis, and severe often difficult to control asthma [41]. AERD has been generally linked to abnormalities in the arachidonic acid biosynthetic pathway where eosinophil infiltration in both upper and lower airways constitute a key feature. However, the exact mechanisms of such chronic eosinophilic inflammation are not fully understood. We have already shown that concentrations of IgE antibodies to SAEs were significantly increased in patients with CRSwNP and AERD compared with controls and CRSwNP without AERD. Furthermore, 54% of AERD patients demonstrated IgE antibody production against SAEs, compared with 26% in the non-AERD and none in the control groups. However, of interest, data from a subgroup analysis for the criterium "IgE antibodies to SAEs" showed that severity of inflammation is related to the presence of IgE to SAE in the group of patients without AERD only [42]. These results were later confirmed by a study demonstrating the increased presence of intracellular *S. aureus *in nasal polyps from AERD patients compared with control nasal mucosa [11]. Additionally, we demonstrated that nasal polyps of patients with comorbid manifestation of asthma and AERD presented a distinct systemic immune response to production and differential expression of effector and T regulatory cell surface markers. SEB induced a significantly higher release of TNF-*α *and IL-2 in nasal polyps from AERD patients after 4 hours of stimulation. In contrast, CRSwNP without AERD and control subjects responded only with an increase of IFN-*γ*. After 18 hours, SEB significantly induced the release of TNF-*α*, IFN-*γ*, IL-2, and IL-5 in both nasal polyp-asthma groups compared with healthy controls but without statistical differences between disease groups [43]. All these data strongly point to *S. aureus *colonization and production of superantigenic toxins as an important modifying factor of the inflammatory process operating in severe asthma and probably AERD. Therefore, adequate management of bacterial infection may help with disease management and control in these patients.

## Possibilities to Counteract the Chronic Inflammatory Process Induced by *S. Aureus*

The first-line treatment strategies of chronic inflammation of the upper airways consist of nasal corticosteroids and antibiotics (with integrated anti-inflammatory activities). Several clinical reports suggested that long-term, low-dose macrolide antibiotics may be effective in treating CRS, which is not curable via corticosteroid treatment [44]. However, in a double-blind placebo-controlled study the effects of methylprednisolone (oral glucocorticoid) and doxycycline (antibiotic) were analyzed. Both significantly decreased nasal polyp size compared with placebo; the effect of methylprednisolone was maximal at week 2 and lasted until week 8, whereas the effect of doxycycline was moderate but present for 12 weeks. Methylprednisolone significantly reduced levels of ECP, IL-5, and IgE in nasal secretions, whereas doxycycline significantly reduced levels of myeloperoxidase, ECP, and MMP-9 levels in nasal secretions [45]. Antibiotics like doxycyclin have only some moderate effects probably because mature bacterial biofilms are able to resist antibiotic concentrations up to 1000 times greater. This is because antibacterial agents have difficulties in penetrating biofilms and killing and/or inhibiting the proliferation of the bacteria within the biofilm [46]. Consequently, antibiotic therapies are generally ineffective for the treatment of biofilm-associated bacterial infections. In case of treatment failure, surgery is indicated. Functional endoscopic sinus surgery (FESS) has become a standard procedure to restore sinus ventilation and drainage by opening the key areas. Symptomatic improvement for nasal polyps after FESS is ranging from 37 to 99% (median 89%) with a 2 to 24% failure rate because of recurrence or bad healing due to persistent inflammation and/or bacterial colonization [47]. Direct targeting of *S. aureus *is another option: one possibility is via immunotherapy against *S. aureus*, which is an attractive goal but until today there is currently nothing available in the clinic [48]. Another possibility is to make use of the destructive effect of bacteriophages on their host organisms, which has been exploited as a strategy for killing infecting bacteria [49]. Recently, the cell wall-degrading enzyme of siphoviridae bacteriophage *φ*11 has been shown to be capable of removing *S. aureus *biofilms and to possess anti-Staphylococcal activity [50,51]. However, effectiveness still needs to be tested in clinical trials.

New therapeutic approaches are focusing on the knowledge of the pathophysiology of nasal polyps: eosinophilic inflammation, T_H_2 cells orchestrating the inflammatory process, and the IgE antibodies produced locally. Anti-IL5 (reslizumab) is a candidate that was already tested in a double-blind placebo-controlled clinical study and induced the reduction of eosinophil numbers and concentration of ECP up to 8 weeks after treatment. However, only the patients with high nasal IL-5 concentration seemed to benefit from this therapy [52]. Another possibility that our group is evaluating is the antagonism of IgE-mediated inflammation because in CRSwNP a high correlation between IL-5 and IgE was found. Superantigens produced locally in the airways may lead to class switching of local B cells, resulting in polyclonal IgE production in the airways and also specific IgE against the superantigen. On the basis of these observations, anti-IgE (Omalizumab) treatment could suppress the IgE-mediated inflammatory cascade in nonallergic diseases such as CRSwNP [53].

## Conclusion

Persistent inflammation of the airways is a disabling disease with high morbidity, especially once asthma has developed. A T_H_2-bias and *S. aureus *enterotoxins play an important role in this inflammatory process. More thorough knowledge regarding the impact of nasal pathogens on structural, innate, and adaptive immune cells is a prerequisite in the process of unraveling the starting phase of the chronic inflammatory process and might possibly help to develop specific therapies.
